# Beyond Sperm and Male Accessory Gland Proteins: Exploring Insect Reproductive Metabolomes

**DOI:** 10.3389/fphys.2021.729440

**Published:** 2021-10-07

**Authors:** Francesca Scolari, Fathiya M. Khamis, Diana Pérez-Staples

**Affiliations:** ^1^Institute of Molecular Genetics (IGM)-CNR “Luigi Luca Cavalli-Sforza”, Pavia, Italy; ^2^International Centre of Insect Physiology and Ecology (icipe), Nairobi, Kenya; ^3^Instituto de Biotecnología y Ecología Aplicada (INBIOTECA), Universidad Veracruzana, Xalapa, Mexico

**Keywords:** metabolomics, mating, ejaculate, seminal fluid, female post-mating response, mass spectrometry, nuclear magnetic resonance

## Abstract

Insect seminal fluid, the non-sperm component of the ejaculate, comprises a variegated set of molecules, including, but not limited to, lipids, proteins, carbohydrates, salts, hormones, nucleic acids, and vitamins. The identity and functional role of seminal fluid proteins (SFPs) have been widely investigated, in multiple species. However, most of the other small molecules in insect ejaculates remain uncharacterized. Metabolomics is currently adopted to deepen our understanding of complex biological processes and in the last 15years has been applied to answer different physiological questions. Technological advances in high-throughput methods for metabolite identification such as mass spectrometry and nuclear magnetic resonance (NMR) are now coupled to an expanded bioinformatics toolbox for large-scale data analysis. These improvements allow for the processing of smaller-sized samples and for the identification of hundreds to thousands of metabolites, not only in *Drosophila melanogaster* but also in disease vectors, animal, and agricultural pests. In this review, we provide an overview of the studies that adopted metabolomics-based approaches in insects, with a particular focus on the reproductive tract (RT) of both sexes and the ejaculate. Progress in the field of metabolomics will contribute not only to achieve a deeper understanding of the composition of insect ejaculates and how they are affected by endogenous and exogenous factors, but also to provide increasingly powerful tools to decipher the identity and molecular interactions between males and females during and after mating.

## Introduction

During mating, males are known to transfer to females not only their spermatozoa, but also non-sperm components in the ejaculate. The proteins within the seminal fluid (SF), regarded as the seminal fluid proteins (SFPs), have been widely characterized, initially in *Drosophila melanogaster* and later in several other insect species (see Meuti and Short, 2019 for a review). The non-protein fraction of the ejaculate includes hormones (e.g., 20E), nucleic, amino and fatty acids, lipids [e.g., the prostaglandins (PG) in crickets], carbohydrates, terpenoids, and defensive compounds (Poiani, 2006; Perry et al., 2013; Hopkins et al., 2018). Both SFPs and non-protein molecules have been shown to have a wide range of physiological and behavioral effects when interacting with female reproductive tissues (Avila et al., 2011), as compounds from both groups, including PGs and steroid hormones, are able to decrease sexual receptivity and stimulate oviposition in mated females (Stanley and Kim, 2011; Worthington et al., 2015). Although hundreds of SFPs have been identified, we are still limited in our understanding of the smaller (<1,500Da; German et al., 2005) molecules of the non-protein fraction of the ejaculate that make up the male reproductive metabolome (RM). These compounds are likely involved in long-term sperm storage, sexual receptivity, and, ultimately, reproductive success and sexual conflict. Ejaculate proteins and the RM are likely interacting, as shown by their concerted antimicrobial role in conferring protection from diseases in the honeybee, *Apis mellifera* (Peng et al., 2016). The RM comprises molecules with various physical and chemical properties that can be delivered to females during mating bound to sperm or packaged into vesicles (Hopkins et al., 2017). This implies that different methods need to be adopted to identify and measure specific RM components, and the respective results have to be integrated.

Metabolomics, i.e., the comprehensive analysis of the metabolites in a system (Fiehn, 2002; Kamleh et al., 2009a), is a powerful tool to (i) identify and measure metabolites in insect organs and biological fluids (e.g., hemolymph and ejaculates), (ii) characterize the metabolic state of a tissue/organ, and (iii) provide a global overview of the changes associated with insect physiology (e.g., development, diet, mating, and aging). Metabolomics is thus instrumental to provide information that complements the genomic, transcriptomic, and proteomic profile of the reproductive system and allows the identification of key molecules involved in metabolic pathways essential to insect reproduction and to other fundamental biological roles.

The aim of this mini-review is to provide a summary of published studies on metabolomics of insect reproductive tissues and ejaculates. We will describe the methodological features of metabolomics-based experiments, the different technological resources available as well as the findings achieved so far. Finally, we will focus on the future directions and challenges of this research field, both in terms of basic and applied science.

## Technical Approaches and Challenges of Insect Metabolomics

Metabolomics-based approaches require the optimal integration of several different experimental and analytical components, namely (i) efficient sample collection to obtain a large number of replicates to cover the maximum possible variation, (ii) sample preparation protocols, (iii) chemical analyses, and (iv) data processing and bioinformatics.

Metabolomics studies can be performed through either targeted or untargeted approaches (Almontashiri et al., 2020). Targeted metabolomics studies focus on the identification of pre-defined chemically characterized compounds. This approach requires sample preparations optimized to select the physiochemical properties of the specific compounds of interest, followed by analysis with chromatography and/or mass spectrometry. This strategy relies on an *a priori* hypothesis and intrinsically identifies a limited set of metabolites. An untargeted metabolomics approach is usually adopted to trace a global profiling of all the metabolites present in a sample, producing a “metabolic fingerprint.” To achieve this goal, it is essential to (1) integrate data from different metabolic profiling technologies with comprehensive chemical information from complex mixtures and (2) process the acquired data with powerful bioinformatic tools, annotate metabolites, perform statistical analyses, identify metabolic pathways, and integrate metabolomics with other omics data.

The first aspect of any accurate metabolomic study is the collection of the samples, which have to be processed and frozen in liquid nitrogen. A rapid and efficient sample homogenization in an organic solvent is essential to release the intracellular metabolites, precipitate the proteins, and terminate the metabolic reactions. Given that metabolite turnover is fast, the sample collection/processing needs to be optimized to stop/quench the enzymatic reactions immediately. The final metabolite measure has to take into account sample loss and different extract efficiencies. Frozen samples can be stored at −80°C, but a fast analysis is recommended to avoid instability of specific metabolites over time. Importantly, several rounds of analysis using different biological samples collected in independent experimental replicates should be carried out (Cox et al., 2017; Onyango et al., 2020). Metabolomics can be influenced by a range of pre-analytical factors (e.g., sample collection, aliquoting, transport, storage, and thawing) and standardizing these steps is essential particularly for field samples, for which handling time may be longer. Moreover, in the case of field samples, quantitative environmental data and molecular profiles should be associated with the biochemical properties revealed by metabolomics in a multi-omics and modeling frame to obtain more robust data (Nagler et al., 2018).

The two most common analytical platforms used in metabolomics studies are mass spectrometry (MS) analyzers (combined with gas or liquid chromatography – GC or LC, but also capillary electrophoresis, CE) and nuclear magnetic resonance (NMR) spectrometers (Soga et al., 2003; Kamleh et al., 2009a; Cox et al., 2017). NMR spectroscopy has been applied for the analysis of metabolic data since the 1970s (Wilson et al., 1974) and strongly contributed to launching the field of metabolomics (German et al., 2005; Wishart, 2008). MS and NMR are complementary due to their respective strengths and weaknesses, resulting in different sets of metabolites that can be detected. While NMR detects the most abundant metabolites in a sample, MS detects those that are readily ionizable (Bhinderwala et al., 2018). NMR-based techniques are robust, fast, reproducible, high-throughput, and non-destructive, but are less sensitive than MS, associated with a higher instrument cost, not optimal for targeted analyses, and allow the identification of a smaller set of metabolites. MS-based methods are associated with lower reproducibility, require more complex sample preparation and tissue extraction, and have longer analysis than NMR and higher costs (Amberg et al., 2017; Miggiels et al., 2019; Pinu et al., 2019).

The analysis of raw metabolomics data consists of the extraction of signals that correspond to individual chemicals and is organized in the following steps: (1) extraction of the chromatographic peaks, i.e., detection and extraction of peaks at specific m/z values, within a defined mass tolerance, and with their retention time (RT); (2) RT alignment to facilitate sample comparison by adjusting RT shifts between injections; (3) deconvolution/componentization based on RT similarity and peak shape to reduce data complexity; and (4) data filtering to eliminate baseline noise. These steps are critically described in several articles (Worley and Powers, 2012; Saccenti et al., 2014; Yi et al., 2016; Cox et al., 2017), and their analysis goes beyond the scope of our review.

The final step in a metabolomics study is the interpretation of the produced data to determine whether observed changes in the metabolite profiles are associated with metabolic pathways. This can be achieved using different online databases, such as Metaboanalyst, The Kyoto Encyclopedia of Genes and Genomes (KEGG), and Reactome (see Cox et al., 2017 for a detailed review). Moreover, different mathematical and computational methods are available for integrating gene expression and metabolomics data (McIntyre et al., 2020; Jendoubi, 2021; Misra, 2021).

Untargeted metabolomics (“discovery metabolomics”), while holding the potential for finding new key biomarkers, has a drawback: the identification of chemicals with mass features not matching any compounds in the available databases, which may limit the discovery of specialized compounds playing key biological roles in target species (Al-Wathiqui et al., 2016). Indeed, *de novo* identification of a compound not having an accessible fragmentation pattern and not confidently identifiable by spectral library searches is particularly challenging and frequently regarded as one of the most relevant bottlenecks in metabolomics. Thus, several approaches have to be integrated, including elemental structure identification and mock fragmentations of candidate structures, with particular attention devoted to functional groups that are likely to fragment and undergo complete ionization (Gertsman and Barshop, 2018). Moreover, one of the biggest analytical challenges is to separate and identify very similar metabolites (e.g., isomeric and isobaric) with different biological functions. To overcome this issue and improve identification, strategies are being developed to verify retention behavior by updating retention information between different experiments allowing smaller tolerance for RT shifts, which decrease the power of compound databases (Opialla et al., 2020).

## Initial Studies of Insect Metabolomes

Nuclear magnetic resonance-based metabolomics had been initially applied in *Drosophila* species to trace metabolic profiles in hypoxic conditions (Feala et al., 2007) and to address questions such as those related to stress resistance (Malmendal et al., 2006, 2013; Feala et al., 2007; Overgaard et al., 2007; Coquin et al., 2008; Pedersen et al., 2008; Cox et al., 2017), aging (Mishur and Rea, 2012; Kapranas et al., 2016), and the effects of pesticides and pollutants on insect biochemistry (Yan et al., 2018). The first applications of MS-based metabolomics in insects (Latocha et al., 2000; Ueyama et al., 2005; Fyrst et al., 2008) included the metabolomic profiling of mutants, including *rosy* (Kamleh et al., 2008, 2009b; Bratty et al., 2011).

In the last decade, GC–MS methods have been applied to address effects of stresses, including heat, freezing, and desiccation (Michaud and Denlinger, 2007; Robert Michaud et al., 2008; Koštál et al., 2011; Colinet et al., 2012; Mamai et al., 2014; Cox et al., 2017; Horvath et al., 2021). Nanospray ionization tandem mass spectrometry approaches have been used to investigate ammonia and nitrogen metabolism in *Aedes aegypti* (Scaraffia et al., 2006, 2008), as well as the changes in metabolomic profiles in response to different diets and after pathogen infection in *Anopheles* (Champion et al., 2017). A GC-TOF/MS system was employed to characterize the global metabolic changes induced by irradiation in the Japanese pine sawyer, *Monochamus alternatus* (Qu et al., 2014), showing the potential of metabolomics as a tool to acquire information on the mechanisms underlying radiation-induced sterility, with wide implications for pest control. In insects, metabolomics studies are exponentially increasing (Snart et al., 2015), particularly in the last 5years.

## Advanced Cross-Platform Data Integration

Currently, the analytical challenges in metabolomics include the need to integrate data derived from more than one platform to produce more robust data and a wider metabolome coverage, maximizing the detection of possible metabolites in the target system (see Aliferis and Jabaji, 2011; Ćeranić et al., 2020; Roca et al., 2021 for reviews). Recent studies are showing that this integration is particularly powerful. For example, Zhou and colleagues combined UPLC-QTOF-MS and GC-Q-MS to explore the metabolic changes induced by azadirachtin, the main active component of neem-based pesticides, on the larvae of the tephritid fruit fly *Bactrocera dorsalis* (Zhou et al., 2020). Another recent study provided a comprehensive analysis of the changes in the whole-body metabolome of the tiger mosquito *Aedes albopictus* after infection with the Zika virus (Onyango et al., 2020) through integrating GC-TOF for identification of primary metabolites (e.g., carbohydrates, proteins, amino acids, vitamins, and hydroxyl acids), LC/Q-TOF CSH for lipid profiling, and HILIC LC/Q-TOF for analysis of biogenic amines (e.g., monoamine neurotransmitters and polyamines). The hot topic of the biochemical response to arbovirus infection and pathology in insect vectors recently included metabolomics as a powerful tool to be applied to both cell lines (Perera et al., 2012; Melo et al., 2016; Bottino-Rojas et al., 2019) and insect tissues (Chotiwan et al., 2018; Shrinet et al., 2018). These studies are contributing to achieve a deeper understanding of the metabolic environments in mosquito tissues where viral infection, replication, and dissemination occur. These findings are essential to identify biochemical markers that could be targeted to interfere with arbovirus infection, thus expanding the toolbox for vector control. Similarly, studies on metabolic fingerprints between hosts and herbivores may lead to the development of new pesticides (Riach et al., 2019; Sanchez-Arcos et al., 2019).

## Metabolomics Applied to the Characterization of Insect Reproductive Metabolites

While a majority of the first insect metabolomics studies were based on a “whole-organism homogenate” approach, thereafter the importance of tracing tissue-specific metabolomes began to emerge (Carvalho et al., 2012; Chintapalli et al., 2012, 2013). These seminal studies showed that the metabolomes of each tissue strongly differ and were also significantly divergent from the data obtained from whole-body analyses (Chintapalli et al., 2013). This finding opened new avenues to better understand physiological processes including diapause (Lu et al., 2014), as well as profiling the metabolites present in the hemolymph (Killiny et al., 2017). However, to date, only a handful of studies focusing on the metabolic composition of insect reproductive tissues are available (Table 1). Interestingly though, these studies are showing the feasibility of performing metabolomics analysis on small amounts of insect tissues and organs.

**Table 1:** Metabolomics studies performed on insect reproductive tissues and ejaculates.

Species	Organs/Ejaculate	Metabolomics platform	Biological question addressed	Reference
*Photinus pyralis*	Spermatophore	LC-HRAM-MS	Characterize synthesis, composition, and fate of the spermatophore	Al-Wathiqui et al., 2016
*Drosophila melanogaster*	mAGs, testes, and ovaries	ZIC-HILIC LC-Orbitrap MS	Explore the metabolite composition of individual tissues	Chintapalli et al., 2013
*Culex pipiens*	mAGs	NMR	Evaluate the differences in the metabolome among adult males reared on different diets	Huck et al., 2021
*Apis mellifera*	Spermatheca	LC–MS	Assess changes in spermathecal metabolome between virgin and new-laying queens	Liu et al., 2020

mAGs, male accessory glands; LC-HRAM-MS, liquid chromatography hyphenated with high-resolution accurate mass spectrometry; ZIC-HILIC LC-Orbitrap MS, zwitterionic–hydrophilic interaction chromatography liquid chromatography–Orbitrap mass spectrometry; NMR, nuclear magnetic resonance; and LC–MS, liquid chromatography–mass spectrometry.

### Metabolomics of the Male and Female Reproductive Organs

Studies tracing the metabolome of male reproductive organs play a key role in (1) determining the relative contribution of testes (T), male accessory glands (mAGs), and other secretory tissues in the production of ejaculate metabolites and (2) investigating phenomena such as oxidative stress and infertility, similarly to what has been done in mammals (Aitken and Baker, 2020; Ribas-Maynou and Yeste, 2020). Only two studies so far adopted a metabolomics approach to this purpose. The first was done in *Drosophila*, and testes, mAGs, and ovaries were included among the organs for which a metabolomic profile was traced (Chintapalli et al., 2013). The testes showed high levels of glutathione, which may provide protection against oxidative stress, and of phosphoarginine, carnitine, and acetyl carnitine, important for sperm motility. The lipid pattern in these organs revealed that phosphatidylethanolamine, phosphatidylcholine, and ether lipids are particularly abundant, reflecting the important role of lipids as sperm membrane components (Chintapalli et al., 2013). The mAGs metabolome showed uniquely high levels of methionine derivatives, such as S-adenosylmethionine, putatively involved in polyamine biosynthesis. The ovary metabolome suggested high metabolic activity, with pathways related to antioxidant defense, stem cell formation, lipid and terpenoid biosynthesis, and energy reserve being particularly represented (Chintapalli et al., 2013).

The second study focused on the metabolomic analysis of *Culex pipiens* mAGs and revealed that the adult male diet had an impact on the composition of these organs, likely affecting SF composition (Huck et al., 2021). By integrating fecundity assays and metabolic results, the authors showed that nutritionally deprived males divert metabolic resources toward energy metabolism instead of reproduction. In particular, NMR spectroscopy allowed the identification of formic acid, which may enhance sperm motility, glucose, lactic acid, fatty acyls, and triglycerides, which were positively correlated with dietary sugar content (Huck et al., 2021). Interestingly, both glucose and lactic acid were detected in the male reproductive tissues of mammals (Mumcu et al., 2020), suggesting a functional conservation that deserves further exploration.

### Metabolomics of Insect Ejaculates

So far, only one study focusing on the metabolomic characterization of an insect male ejaculate has been published. Specifically, this study aimed at characterizing the metabolome of the spermatophore in the common Eastern firefly *Photinus pyralis* (Al-Wathiqui et al., 2016). Spermatophores are sperm-containing capsules composed of secretions derived from the mAGs (Lewis et al., 2014) and also from the testes, as shown in the tsetse fly *Glossina m. morsitans* (Scolari et al., 2016). Al-Wathiqui et al. (2016) applied a multi-omics approach integrating MS/MS proteomics and liquid chromatography hyphenated with high-resolution accurate mass spectrometry (LC-HRAM-MS) metabolomics to characterize the spermatophore components in *P. pyralis*. In addition to 208 proteins, an untargeted metabolomics analysis found that several mass features were exclusively present (or present at significantly higher abundance) in the spermatophore, further supporting the idea that targeted and untargeted metabolomics are not mutually exclusive but can be profitably integrated. Given the absence of matches to any compounds in the KEGG database, some of these chemicals may be specialized molecules yet to be identified. Using a targeted metabolomics analysis, lucibufagin, a known firefly defensive toxin, was detected in both the spermatophore and the male body (Al-Wathiqui et al., 2016). Similarly, in the beetle *Neopyrochroa flabellate*, defensive terpenoids (cantharidin) have been found to be ingested by males, stored in the mAGs, and then transferred and used by females for egg defense (Eisner et al., 1996). Therefore, metabolomics will help us understand the evolution of ejaculate nuptial gifts that may protect females and/or eggs against predators or microbial attacks.

### Metabolomics of Female Sperm Storage Organs

Analysis of the sperm storage organs holds great potential as it has implications for the rearing and conservation of beneficial insect species, such as honeybees, and in the optimization of strategies for the control of pest and vector populations in the wild. Indeed, by unravelling the physiological mechanisms underlying sperm storage, it may be possible to develop tools to affect fertility, as well as expand our knowledge on oogenesis. Furthermore, by comparing metabolites in reproductive tissues between unmated and mated females and males, it may be possible to discern among metabolites produced, received, stored, and potentially used by females. Similarly to males, females produce proteins in their reproductive tract (RT) that mediate inter-sex molecular interactions (Schnakenberg et al., 2011; Sun and Spradling, 2013). Expanding knowledge on the small molecule set produced in female RT will contribute to further understanding the interactions between sexes. To assess the post-mating metabolic changes occurring in the spermatheca of *Apis mellifera*, an LC-MS analysis was performed to compare the metabolites of virgin (VQS) and new-laying queens (NLQS; Liu et al., 2020). Of the total 7,745 metabolites identified, 861 were found to be differentially expressed, with 384 being significantly higher in their abundance in NLQS, and 477 significantly lower. Authors detected changes in lipids and lipid-like molecules during sperm storage in NLQS, such as fatty acyls, glycerophospholopids, prenol lipids, and sterol lipids. These data are particularly relevant as they highlight key target metabolites and biosynthetic pathways with potential important roles in sperm storage and reproductive success.

### Applications of Insect Metabolomics: Knowledge Gaps and Future Trends in Reproductive Biology

**Figure 1:**
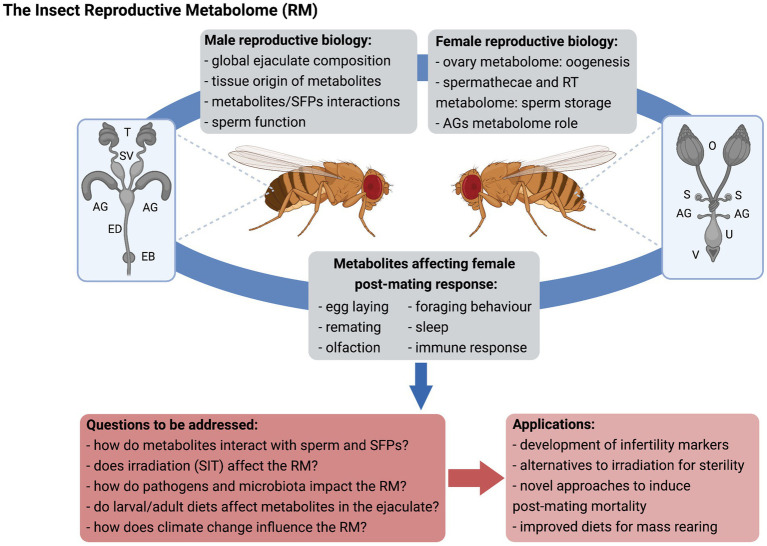
The insect reproductive metabolome (RM). Metabolomics can be applied to the study of male and female reproductive biology and expand knowledge on the physiological mechanisms underlying inter-sexual interactions upon mating, with relevant practical application for insect control. SFPs, seminal fluid proteins; RT, reproductive tract; AG, accessory glands; T, testes; SV, seminal vesicles; ED, ejaculatory duct; EB, ejaculatory bulb; O, ovaries; S, spermathecae; U, uterus; V, vulva; SIT, sterile insect technique; and RM, reproductive metabolome. Figure created with BioRender.com.

From a basic science perspective, the characterization of RMs will allow (i) identification of key players supporting sperm function, storage and use, and female fertility, (ii) evaluation of the impact of insect physiology and the environment on ejaculate composition, (iii) a better understanding of the respective contribution of specific male and female organs (Figure 1), and (iv) an expanded knowledge on how male-derived molecules interact with the female RT, particularly beyond SFPs. The RM will provide insights into ejaculate evolution, so that production costs of these complex molecules and fitness trade-offs can be determined and compared across species. Identification of non-protein components of the ejaculate, such as PGs, terpenes, and sexually transmitted pathogens is critical to understand how males and females may be under differential selection pressures on immune response, and regulation of remating rates to name a few.

Metabolomics may help in expansion of knowledge and management approaches for controlling agricultural and animal insect pests, as well as vectors of multiple diseases. From the applied point of view, metabolomics can contribute to the fingerprinting of the ejaculates produced by irradiated males in the context of the sterile insect technique (SIT; where the target species is mass-reared, sterilized, and released so males will mate with wild females transferring lethal dominant mutations in their sperm and thus avoiding offspring production; Dyck et al., 2021). This will allow for a novel and sophisticated male quality control and, more specifically, for the identification of markers of infertility, similar to what has been achieved in mammals (Zhao et al., 2018; Engel et al., 2019; Menezes et al., 2019; Murgia et al., 2020), to determine SF fertilizing capacity. The availability of these markers may help in identifying the metabolic pathways producing such key metabolites and will also enable identification of related gene candidate(s) that can be manipulated to either (i) explore strategies alternative to irradiation to achieve sterility, (ii) find pathways that could be targeted in the female to induce mortality after mating with manipulated males, and/or (iii) increase the fecundity and fertility of mass-reared insects.

Metabolomics is particularly promising also to investigate the metabolites produced by the gut microbiota that may have an impact on the quality of mass-reared insects, in relation to different types of larval and adult diets. This exciting area of research may lead to the identification of compounds to be exploited as probiotic supplements in larval diets and deciphering the functions of insect host–bacterial symbiont interactions. Bing et al. (2017) explored the metabolic co-evolution between *G. m. morsitans* and its obligate endosymbiont *Wigglesworthia* using a combined transcriptomics and metabolomics approach. The absence of the symbiont-derived B vitamins affects tsetse fly metabolism, physiology, and survival in multiple ways and supports the key role of this symbiont in ensuring the fitness of the fly host. Although metabolomics studies are increasingly being undertaken, this field is ripe for future studies combining transcriptomics, proteomics, and metabolomics.

Such an integrated approach can now be applied to studies comparing pre- and post-mating changes in females and males. Moreover, the integration of metabolomics with up-to-date metagenomics analyses can elucidate the role of symbionts in insect ejaculates. Indeed, insect ejaculate is not “sterile,” but it is known to be populated by microbes and viruses (Poiani, 2006; Otti, 2015; Rowe et al., 2020). Several metabolic pathways triggered by mating and/or by the symbionts present in the male ejaculate may be revealed, thus opening novel research horizons.

## Author Contributions

FS conceived the topic for the mini-review and prepared the figure. FS, DP-S, and FMK wrote the manuscript. All authors contributed to the article and approved the submitted version.

## Conflict of Interest

The authors declare that the research was conducted in the absence of any commercial or financial relationships that could be construed as a potential conflict of interest.

## Publisher’s Note

All claims expressed in this article are solely those of the authors and do not necessarily represent those of their affiliated organizations, or those of the publisher, the editors and the reviewers. Any product that may be evaluated in this article, or claim that may be made by its manufacturer, is not guaranteed or endorsed by the publisher.
